# Differential enrichment of regulatory motifs in the composite network of protein-protein and gene regulatory interactions

**DOI:** 10.1186/1752-0509-8-26

**Published:** 2014-02-27

**Authors:** Shubhada R Hegde, Khushbu Pal, Shekhar C Mande

**Affiliations:** 1 Centre for DNA Fingerprinting and Diagnostics, Bldg 7, Gruhakalpa, 5-4-399/B, Nampally, Hyderabad 500 001, India; 2 Current Address: National Centre for Cell Science, NCCS Complex, University of Pune Campus, Ganeshkhind, Pune, Maharashtra 411007, India; 3 Current Address: Cellworks Research India Ltd, Bangalore, India

**Keywords:** Protein-protein interaction, Gene expression, Motifs, Network, Gene regulation

## Abstract

**Background:**

An important aspect of molecular interactions is the dynamics associated with growth conditions. Intuitively, not all possible interactions take place together all the time in a cell as only a subset of genes is expressed based on environmental conditions.

**Results:**

Large scale gene expression data of *Escherichia coli* was analyzed to understand the dynamics exhibited at expression level. A large compendium of gene expression datasets, which covers about 466 growth conditions, was used for the analysis. Using gene expression data, genes of *E. coli* were profiled into three classes: Widely expressed, Conditionally expressed and Rarely expressed. Further, dynamics associated with molecular interactions were analysed by studying changing importance of motifs in the composite networks across growth conditions.

**Conclusions:**

Our analysis of large scale gene expression data suggests conditional expression of genes which brings about befitting responses for a given growth environment. We observe a range of importance for network motifs across conditions which can be correlated with a specific function. Our study therefore suggests rewiring of molecular interactions driven by gene expression changes depending on the conditional needs.

## Background

Properties of complex systems are believed to be characterized by a network of interactions among components of the system. An intricate balance of interactions exists among the different components, which determines the manner in which the system responds to perturbations. Cells respond to perturbations in environmental conditions by means of dynamics of interactions among different proteins in the cells, and by means of changes in the gene regulatory circuits. The latter lead to changes in global gene expression, which are amenable to experimental studies, and indeed many experimental studies have been able to map the response of gene expression under different environmental conditions. The effect of the former is, however, not understood in great experimental depth. Moreover, mapping genome-wide protein-protein interactions is experimentally demanding technique, making it difficult to sample interactomes under different conditions and study their dynamics.

At the systems level, complex interplay of interactions can be represented in the form of interaction maps [1]. Graph theoretical analysis on protein interaction networks enables understanding of gene essentiality, modular organization of functional pathways and protein function [2]. In this regard, analysis of the dynamic profile of protein interactions in *E. coli* was employed to understand cellular responses upon UV treatment [3]. Similarly another study identified modules of protein interactions with different network topologies in *S. cerevisiae* by integrating protein interactions with gene expression [4]. Such studies highlight the importance of protein interactions in the context of varying growth conditions or genotypes. Since global properties of the profile of interactions representing each condition are unlikely to differ due to system robustness, a better way has been proposed by us is to examine the dynamics at local structural and functional units of the network [3]. One such local measure is the network motif in a regulatory network, which is a structural unit that appears more frequently in the real network than in randomized networks indicating a functional importance [5]. Motifs have been widely studied in gene regulatory networks, food webs, electronic circuits and other real-world [5]. Interestingly, these patterns have been analyzed to perform specific information processing functions enabling regulated cellular responses [6]. Also, in order to capture the complexity of molecular interactions in a cell, motifs were identified in a composite network comprising of both protein:protein and regulatory interactions [7]. It is therefore important to understand how the significance of a motif varies in a given growth condition or a genetic makeup as large sets of interactions emerge or disappear conditionally.

In an organism, such dynamic rewiring of molecular interactions is accomplished by regulating gene expression. Therefore, in order to establish cellular responses to a spectrum of growth conditions, it is relevant to address how gene expression is regulated dynamically. DNA microarrays have been used to quantitatively describe gene expression [8]. It allows for the global measurement of mRNA transcripts in a cell. With technological developments, introduction of novel algorithms for data analysis and the availability of tools and software, microarray technology has found widespread application in biological research [9,10]. Organized public databases thus became inevitable to accommodate increasing amount of expression datasets in number of organisms [11]. In this regard, databases such as NCBI-Geo [12], MMMD [13] and ArrayExpress [14] function as repositories for individual experiments carried out across laboratories. These databases facilitate a user to access data in large scale and perform genome-wide studies.

In this work, we have studied large scale expression data to understand the dynamics of gene expression in *E. coli*. On a global scale, we have categorised genes based on their expression across growth conditions and studied properties of these classes in terms of mRNA half life, network centrality and conservation. Further, it is important to understand how changes in gene expression are translated to rewiring of molecular interactions. We characterized integrated protein:protein interaction and gene regulatory networks in terms of significance of motifs under different expression conditions. The changing patterns of enrichment of network motifs in these networks were studied to understand the dynamics of molecular interactions. We broadly test if network motifs are conserved under all conditions, or are enriched in certain specific conditions only.

## Methods

Expression data was downloaded from Many Microbe Microarray Database [13] (http://m3d.mssm.edu/) which consists of expression information for 4297 genes of *E. coli* in 466 growth conditions. We have used SpeCond tool to identify conditionally expressed genes in our datasets [15]. SpeCond implemented in Biocondutor package was run with default parameters. Further, in order to determine whether a gene is expressed in a given condition, the median was calculated for the distribution of expression intensities of all the genes in a condition. A gene *i* with expression intensity *X*_*i*_ is considered expressed in condition *j* if *X*_*i*_*/ Median*_*j*_ > 1, as employed in [3]. Using this criterion, a binary profile denoting the presence or absence of the genes of *E. coli* across 466 growth conditions was constructed. Essential genes and non-essential genes were obtained from KEIO collection [16] and Posfai *et al.*[17] respectively. We observed that the average number of conditions in which essential or non-essential genes is expressed to be 89% and 29% respectively. A gene is therefore classified as ‘Widely expressed’ if it is expressed in more than 89% conditions, ‘Rarely expressed’ if the expression is in less than 29% conditions and ‘Conditionally expressed’ otherwise. To test if the cutoff used for gene classification is introducing any bias, two other cutoffs, descriptively, *X*_*i*_*/ Median*_*j*_*>* 0.9 *and X*_*i*_*/ Median*_*j*_*>* 1.1 were used, which yielded similar results. However, changing this cut-off by one order of magnitude (median × 10 or 0.1) results in profiles where either all genes are repressed or all genes are expressed. Therefore, we believe that these cutoffs become too stringent to classify genes as either expressed or not expressed.

In the functional linkages network predicted using genome-context methods [18], top 30% high degree nodes are defined as hub proteins. Phyletic retention was calculated by bi-directional blast of *E. coli* protein sequences against 362 bacterial genomes with e-value cutoff of 1e-04^.^ The data for mRNA half-lives were obtained from Bernstein *et al.*[19] and Selinger *et al.*[20]. Orthologs of *Mycoplasma genitalium* were identified using bi-directional blast with e-value cutoff of 1e-04. Network centrality measures were calculated according to [10]. Pathway classification for *E. coli* genes were downloaded from KEGG database [21].

Composite interaction network for *E. coli* was made by merging protein-protein interactions and gene regulatory interactions. Protein interactions were taken from predicted genome-wide protein functional linkages [18]. Gene regulatory interactions were downloaded from RegulonDB database [22]. In total, about 81176 unique molecular interactions were derived upon combining these two types of interactions. Protein interactions are represented with bi-directional edge and the regulatory interactions are represented as directed edge. In cases where there exists both regulatory and protein interaction, the regulatory interaction is taken into consideration. Autoregulatory interactions were excluded in our analysis to simplify motif prediction output for further analysis. In order to focus on the interaction patterns in the vicinity of regulatory circuits, about 262 transcription factors were used as seeds (Additional file 1), and a path length cutoff of 2 from the seed nodes was employed to derive interaction network. Thereby, we hope to eliminate majority of the interactions which include only proteins without the corresponding transcription factors. This final network is comprised of 77495 molecular interactions (Additional file 2).

Conditional networks were constructed using microarray data. Gene expression data for 466 growth conditions was from Many Microbe Microarray Database [13]. Genes were categorized as ‘expressed’ and ‘not-expressed’ using median expression as the cutoff [3]. While constructing conditional networks, protein interactions were eliminated if the gene corresponding to either of the two proteins is not expressed in the given condition. Motif detection tool FANMOD was used to detect all possible three-node and four-node motifs in each of the conditional networks [23]. Comparison was made with 500 randomized networks to obtain enrichment score for each motif. A motif is considered significant in a given condition if it occurs with a P < 0.05 compared to random networks.

## Results and discussion

### Profiling of *E. coli* genes

In order to categorize presence or absence of the genes of *E. coli* based on expression intensity, publicly available gene expression data was used. Uniformly normalized microarray data for 4297 genes in 466 unique growth conditions was considered for the analysis [13]. There are many methods to score for the expression of a gene using microarray data. SpeCond is one such tool that considers expression intensity distribution and identifies outliers as specifically expressed (conditionally expressed), specifically repressed (conditionally repressed) and the rest as ‘not conditional’ [15]. When we used SpeCond to identify conditional gene expression in our dataset, we identify 62 conditionally expressed genes. Since our data constitutes varied conditions, it is likely that there is a much larger set constituting conditionally expressed genes. Therefore, we have used median expression intensity cutoff as a measure to categorise genes as expressed or not expressed [3]. A gene was therefore scored for its presence (or absence) depending on the median expression intensity in each condition (Methods). Such a profile when obtained for the known essential and non-essential genes in *E. coli* showed expression in about 89% of the conditions for the essential genes and only 29% of the conditions for the non-essential genes. Correlation between essentiality and higher connectivity for proteins in the interaction network is well known [24]. A similar test was therefore performed for the hubs which are defined as highly connected proteins in an interaction network. This analysis revealed that hubs are expressed in 78% conditions. Therefore, based on the number of conditions a gene gets expressed, *E. coli* genes were profiled into three categories: genes that are expressed in majority of the conditions, genes that are expressed only under a few conditions and genes that are rarely expressed. These three classes are named as Widely expressed, Conditionally expressed and Rarely expressed respectively (Additional file 3).

**Figure 1:**
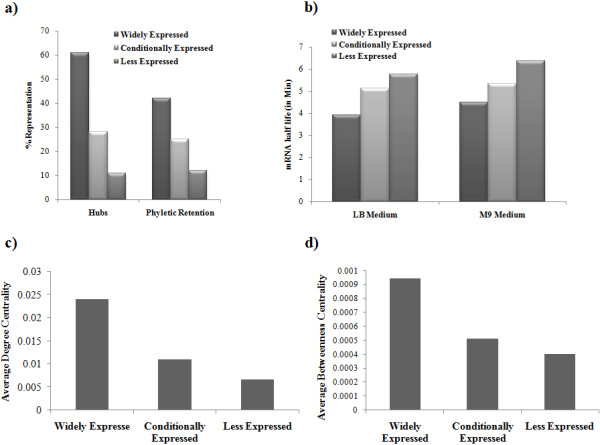
**Properties of gene classes. a)** Widely expressed gene class is enriched for hubs and it is conserved across genomes. **b)** Transcripts of the Rarely expressed gene class are more stable compared to both Widely expressed and Conditionally expressed classes. **c)** and **d)** Genes from the widely expressed class have higher centrality values. Panel **(c)** is for degree centrality and panel **(d)** is for betweenness centrality.

When phyletic retention was studied for these classes, Widely expressed genes were more conserved across genomes compared to Conditionally expressed and Rarely expressed classes (Figure 1(a)). In addition, Widely expressed class was enriched for the orthologs of *Mycoplasma genitalium*, one of the smallest prokaryotic genomes (P-value < 5.09e-0122), suggesting that it consists of proteins from the conserved pathways. About 50% of the hubs are found to be present in the Widely expressed class, reinforcing their essential functions (Figure 1(a)).

In order to test whether the stability of the transcripts from these classes differ, mRNA half-life measurement data in both LB as well as M9 media were considered [19]. Notably, genes of the Rarely expressed class code for more stable transcripts compared to the other two classes (Figure 1(b)). Our definition of presence of a gene in a given condition requires that it expresses more than the median expression intensity. We used additional cutoffs by increasing or decreasing the median cutoff limit to ascertain our observations (Methods). Similar analyses with these cutoffs also yield the same results suggesting that our cutoff used for the classification of genes into different expression classes is unbiased. Therefore, it appears that even though essential genes are transcribed in large amounts, their transcripts are degraded faster, suggesting a faster cellular response in transcription and their tighter regulation.

In order to understand the role of proteins from these three classes, centrality measures in the protein functional linkages were calculated [25]. Proteins coded by Widely expressed genes possess high degree as well as high betweenness centrality followed by Conditionally expressed and Rarely expressed classes (Figure 1(c) and (d)). This implies that Widely expressed genes form the backbone of a functional interaction network and play a critical role in information transfer. The genes from Conditionally expressed class might temporally connect to this core of interacting proteins. Rarely expressed genes, on the other hand, have fewer connections and do not seem to play any significant role in communication within the network.

Analysis of metabolic pathway representation of genes from these three classes revealed interesting aspects of their functional significance. Pathways such as amino acid metabolism (P-value < 2.2 × 10^-16^), nucleotide metabolism (P-value < 0.0011), transcription (P-value < 0.0005) and translation (P-value < 2.2 × 10^-16^) are enriched for Widely expressed genes. On the other hand, genes from Conditionally expressed class are present in higher proportion in cell motility (P-value < 2.2 × 10^-16^) and polyketide metabolism pathways (P-value < 0.04). Pathway enrichment therefore suggests essential cellular functions performed by Widely expressed genes.

### Differential enrichment of motifs

**Figure 2:**
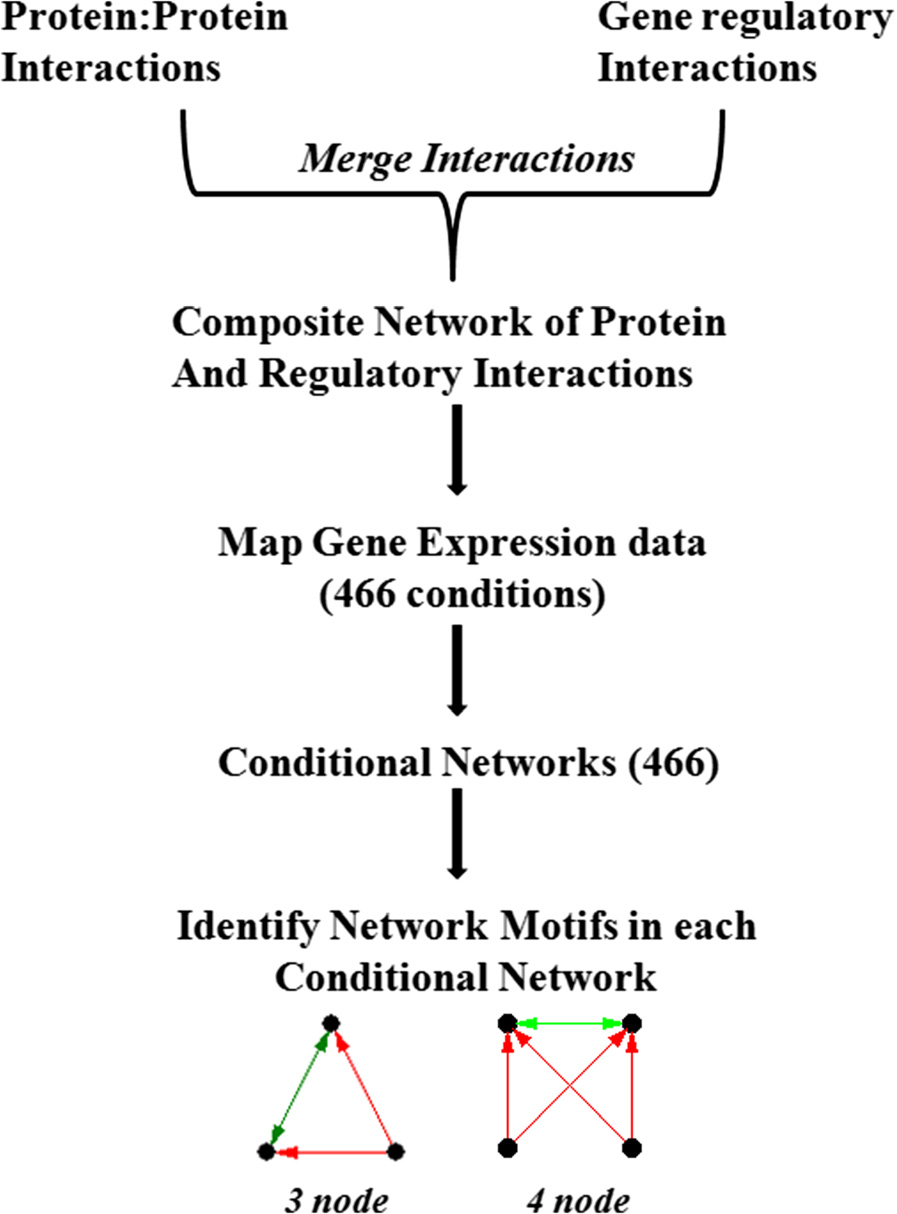
Flowchart depicting the construction of conditional composite networks and motif analysis.

In a cell, regulated gene expression is eventually translated into molecular interactions. The network of such interactions is shown to consist of small sub-structures termed motifs which show specific role in information processing [6]. Since each of these motifs is required for a classified function depending on the conditional needs, our objective was to understand how the importance of such network motifs varies with growth conditions. For this, we have chosen to study this phenomenon in the composite network of protein-protein and gene regulatory interactions. Protein interaction network used in this study is a prediction output based on multiple parameters which includes phylogenetic profile, gene distance and operonic frequency [18]. Biases based on a single prediction parameter are unlikely to exist as these features are effectively combined using Support Vector Machine. In order to construct composite networks representing different growth conditions, we have mapped gene expression data onto a parent network of combined protein-protein interactions and gene regulatory interactions (Methods). We then tested the variability of each of the conditions by counting number of nodes and edges in each of the conditional composite network. As shown in Additional file 4, these networks differ with edges varying from 19000 to 38000 suggesting conditional emergence of interactions. In each of the conditional composite networks, motifs of the sizes three and four were identified. Comparing their occurrence in the random networks, an enrichment score was given to each motif to understand its importance in the given conditional network (Figure 2).

**Figure 3:**
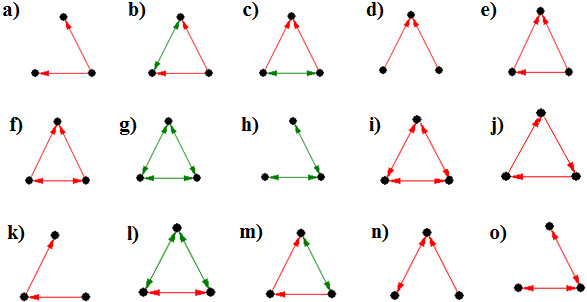
**Some of the enriched 3-node motifs (a-o).** Edges colored in red are the gene-regulatory interactions and those colored in green represent protein:protein interactions. Possible significance of these is described in the text.

**Figure 4:**
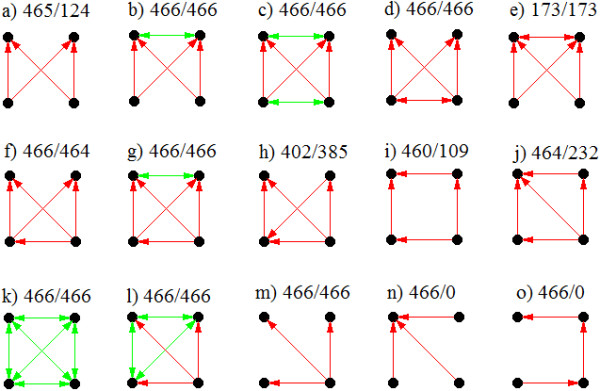
**Few of the enriched 4-node motifs (a-o).** Edges colored in green and red represent protein:protein and regulatory interactions respectively. Numbers denote the condition in which a particular motif is detected and number of conditions it appears significant. These motifs are described in detail in the text.

Considering motifs consisting of purely regulatory interactions, there are about 13 3-node motifs possible. However, this number increases for the interactions consisting of both gene regulatory and protein-protein interactions. There are about 100 possible motif patterns with three nodes and two types of interactions [7]. Whereas 29 motifs were identified in the composite network of *S. cerevisiae*, 22 motifs were observed in the composite network of *E. coli.* Of these 22 three-node motifs, 9 motifs were found to be significant in all the conditional networks. In addition, there are 10 motifs which become significant conditionally. Similarly, with the possibility of >3000 motif patterns considering four nodes and two types of interactions, 323 motifs were detected in the composite conditional networks of *E. coli.* Of these, 60 were significant in all the conditional networks and about 53 motif patterns, though detected, were not significant in any of the conditions. The rest of the 210 motifs were conditionally significant (Table 1). Figures 3 and 4 represent some of the motifs that have been detected, and we describe them briefly below.

**Table 1:** Analysis of 3 and 4 node motifs in conditional composite networks

	**3-node**	**4-node**
Total number of motifs detected	22	323
Significant in all the conditions	9	60
Conditionally significant	10	210
Not significant in any	3	53

#### 3-node motifs

We observe that a pattern in which a transcription factor regulates two genes is a common occurrence in all the conditional networks (Figure 3a). Interestingly, of the 184 transcription factors listed in RegulonDB database [22], only 6 regulate a single gene. Also, motifs where two functionally interacting proteins are regulated by the same transcription factor or two functionally interacting transcription factors regulating the same gene occur frequently (Figure 3b and c). Such a scenario is commonly observed for the operonic gene pairs as they are co-regulated to perform related functions. In addition, it is likely that functionally interacting transcription factors regulate the same gene. Interestingly, pattern where two transcription factors regulating a third gene is insignificant across conditional networks (Figure 3d). However, if the transcription factors share a functional linkage, the motif is significant in all the conditions (Figure 3c).

As reported in earlier studies, we observe that feed-forward motif is significantly enriched in all the conditions (Figure 3e). The importance of such motifs can be illustrated in the transcription of genes involved in iron uptake. Cyclic AMP receptor protein (Crp), which regulates the expression of genes involved in energy metabolism, positively regulates the expression of ferric uptake regulator (Fur). Fur in turn represses the expression of *fec* and *ent* which are involved in iron uptake. Crp additionally regulates these operons positively, thus forming inhibitory-feed-forward loops. Such motifs are studied to be involved in pulse generation and response acceleration [6]. This suggests a cross-talk between availability of carbon source and iron in maintaining homeostasis. Also, a modified feed-forward motif where two transcription factors regulate each other also appears to be important in all the conditions (Figure 3f). Many such regulatory motifs are observed in Crp-Fis regulon where Fis and Crp regulate each other in addition to regulating genes coding for proteins such as D-xylose, maltose and nitrite transporters, and proteins involved in lipid metabolism. Another enriched motif is the protein clique which represents complexes of interacting proteins that work together as multi-component machinery (Figure 3g). Such interactions could be physical as seen in ribosome and transcription assemblies, or functional as observed in biochemical pathways. All these motifs denote essential structures of biomolecular interactions which are independent of growth conditions.

While we do not find two regulatory interactions or two protein interactions at significant threshold in any of the conditional networks (Figure 3d and h), patterns such as closed feedback loops or regulatory cliques, where TFs cross-regulate each other, are not detected in any of the conditions (Figure 3i and j). Closed feed-back loops, though important in electronic circuits, are not preferred by biological systems as they might cause instability and noisy oscillations [5].

Some of the motifs are conditionally important. This set includes two-step input motif (Figure 3k). In 75% of the conditions, the structure with two co-regulating TFs functionally interacting with a third protein is significant (Figure 3l). Whereas two-step input motif is significant only in 9% of the conditions, the same motif closed by a functional interaction is significant in 74% of the conditions (Figure 3m). Interestingly, though the structure where a TF regulates two co-regulated TFs is found in about 345 conditions, they are significant in only about 171 (Figure 3n). Two co-regulatory interactions are observed in 32% of the conditions in which it is detected (Figure 3o). Therefore, varied importance of motifs across growth conditions suggests emerging importance of conditional responses in an organism. A detailed list of motif structures and their conditional significance is provided in Additional file 5.

#### 4-node motifs

Often a gene is regulated by more than one transcription factor as it provides hierarchy of control over gene expression. Therefore, motifs with two transcription factors regulating two genes, termed bi-fan motifs, are studied to be important in regulatory networks [5]. Interestingly in the composite network, simple bi-fan motifs are significantly enriched in only about 25% of the conditions (Figure 4a). Additional interactions emerge in basic bi-fan motifs which are significant in all the conditional networks. Often, two regulated genes in the bi-fan motif are functionally linked (Figure 4b). This exemplifies operonic gene pairs which are regulated by a same set of transcription factors. For example, operon *citCDEFXG* which codes for citrate lyase synthetase, is regulated by transcription factors ArcA, Crp, DipA, FNR and NarL. Notably, in addition to functionally interacting regulated genes, bi-fan motifs with functionally interacting transcription factors are also significant in all the growth conditions (Figure 4c). The motif where regulatory genes in the bi-fan motif are co-regulated with each other is enriched in all the growth conditions (Figure 4d). Also, bi-fan motif where two regulated genes are also co-regulated with each is enriched in about 37% of the conditions (Figure 4e).

A regulatory edge in the basic bi-fan motif results in the overlapping-feed-forward-motif which is significant in almost all the conditions (Figure 4f). Analysis of composite networks reveals that such motifs often regulate genes which are functionally linked. An example of such a motif is observed in arabinose operon where AraC and Crp regulate the expression of *araBAD* operon. Additionally, Crp positively regulates the expression of *araC* forming overlapping feed forward motifs. Interestingly, overlapping feed-forward motif with functionally interacting genes that are regulated is enriched in all the conditions (Figure 4g). Moreover, an inverted edge in the overlapping-feed-forward-motifs is observed in about 402 conditions wherein it is significantly enriched in 385 conditions (Figure 4h).

Another four-node motif is the bi-parallel motif which is detected in most of the conditions. However, the enrichment of such a motif is observed in 23% of the conditions (Figure 4i). Bi-parallel motifs are significantly enriched in neuronal networks, food webs and electronic circuits [5]. Interestingly, structure with a direct edge in the bi-parallel motif is enriched in about 50% of the conditions (Figure 4j). As in the case of three-node motifs, four-node protein cliques are enriched in all the conditions (Figure 4k). Such multi-protein complexes represent functionally interacting modules of proteins. Also, motifs where a transcription factor regulates functionally interacting proteins are enriched in all the conditions (Figure 4l). Also, single input module (SIM) wherein a transcription factor regulates more than one target genes is enriched in all the conditions (Figure 4m). Such interactions possibly represent the regulatory architecture displayed by global regulators which control the expression of genes belonging to diverse pathways. However, a pattern with a gene regulated by multiple transcription factors is not significant in any of the conditions (Figure 4n) suggesting that additional protein-protein or regulatory interactions are common in such situations. Also, as opposed to three-chain motifs which are significantly enriched in food-webs, four-chain motifs, though identified in all the conditions, are significant in none (Figure 4o). Additional file 6 lists the adjacency matrices for the motifs that are identified in conditional networks, total number of conditions they are present and the number of conditions they appear significant.

## Conclusions

It is observed earlier that not all genes are expressed in a given condition in an organism [26]. Genes coding for proteins that perform basic cellular functions are invariably expressed in all the conditions, and are therefore termed as essential genes. In addition, condition specific cellular processes are turned on based on the expression of conditionally essential genes. The other class of genes which is not expressed in most of the conditions is termed non-essential, and they impart redundancy to the system. While experimental profiling of the genes has been carried out previously [16,17], the availability of large-scale gene expression data allows one to perform such studies in a faster and less expensive manner.

We have performed systems level analyses of *E. coli* gene expression by coupling available microarray data with protein interaction network, mRNA half-life and metabolic pathways. *E. coli* genes can be profiled into three classes depending on their expression. The class ‘Widely expressed’ is enriched for hubs and essential genes, and is highly conserved across genomes. The class ‘Rarely expressed’ is less conserved and codes for stable transcripts compared to both Widely expressed and Conditionally expressed classes. Since dynamics in gene expression is eventually translated into molecular interactions, we have chosen to study varying significance of motifs in composite networks across growth conditions. Motifs such as 3-node feed forward loops and bi-fan with protein interactions between regulated genes are significant in all the conditions. On the other hand, closed feed-back loops do not appear to be enriched in any of the networks. It is interesting that not all motifs are equally important in a given growth environment, and new patterns emerge depending on the conditional needs. Therefore, gene expression dynamics can be translated into conditional/context dependent protein interactions which provide useful insights into temporal responses of an organism depending on growth environment.

## Competing interest

The authors declare that they have no competing interests.

## Authors’ contributions

SRH and SCM conceived the study, SRH and KP carried out the analysis, SRH and SCM wrote the manuscript. All authors’ read and approved the final manuscript.

## Supplementary Material

Additional file 1 List of Transcription factors in the composite network. Click here for file

Additional file 2 **Composite network generated by merging protein:protein and regulatory interactions.** Third column represents the nature of interaction (0 for Protein interaction and 1 for regulatory interaction). Click here for file

Additional file 3 **Genes of *E. coli *** **classified into Widely expressed, Conditionally expressed and Rarely expressed classes.** Click here for file

Additional file 4 Distribution of the number of edges (a) and number of nodes (b) in conditional networks. Click here for file

Additional file 5 Complete list of 3-node motifs and their enrichment statistics. Click here for file

Additional file 6 **Complete list of 4-node motifs and their enrichment statistics.** First column is the adjacency matrix for the motif. Second and third columns represent number of conditions the motif is identified and the number of conditions in which it is significantly enriched, respectively. Click here for file
