# Supplementary figures and images for: Differential enrichment of regulatory motifs in the composite network of protein-protein and gene regulatory interactions
Source: BMC Syst Biol. 2014 Feb 27;8:26. doi: 10.1186/1752-0509-8-26 (PMC4015501; doi:10.1186/1752-0509-8-26)

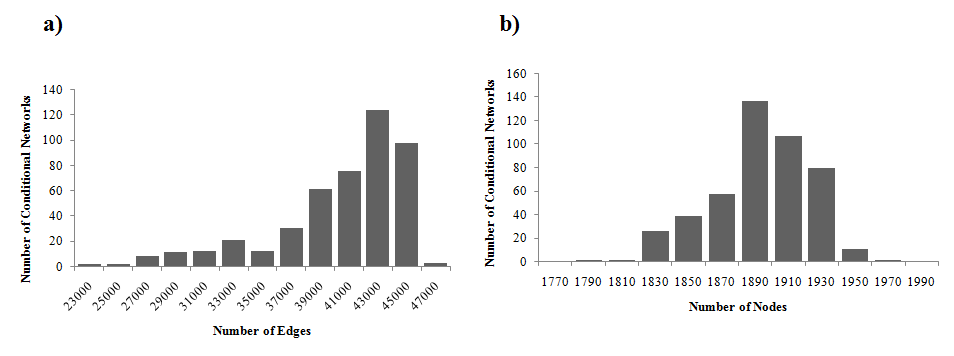

Supplement: Additional file 4 — Distribution of the number of edges (a) and number of nodes (b) in conditional networks. [file 1752-0509-8-26-S4.tiff]
