# Supplementary material for: Differential enrichment of regulatory motifs in the composite network of protein-protein and gene regulatory interactions
Source: BMC Syst Biol. 2014 Feb 27;8:26. doi: 10.1186/1752-0509-8-26 (PMC4015501; doi:10.1186/1752-0509-8-26)
Supplement: Additional file 5 — Complete list of 3-node motifs and their enrichment statistics. [file 1752-0509-8-26-S5.doc]

**Motifs Found in *SN**


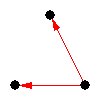
 466


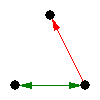
 466


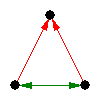
 466


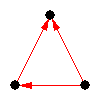
 466


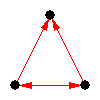
 466


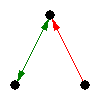
 466


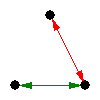
 466


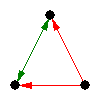
 466


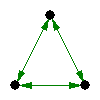
 466

**Motif Id Significance**

6 significant in all

14 significant in all

46 significant in all

38 significant in all

46 significant in all

164 significant in all

78 significant in all

166 significant in all

238 significant in all


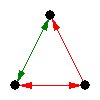
 466


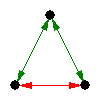
 466


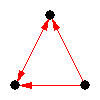
 345


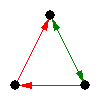
 466


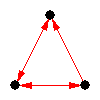
 278


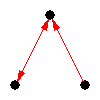
 420


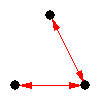
 135


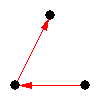
 466


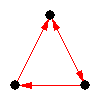
 98

174

| significant - 281 |
| --- |
| insignificant- rest |

238

| significant - 347 |
| --- |
| insignificant - rest |

166

| significant - 171 |
| --- |
| insignificant- rest |

102

| significant - 345 |
| --- |
| insignificant - rest |

174

| significant - 124 |
| --- |
| insignificant - rest |

164

| significant - 84 |
| --- |
| insignificant - rest |

78

| significant - 44 |
| --- |
| insignificant - rest |

12

| significant - 43 |
| --- |
| insignificant - rest |

102

| significant - 4 |
| --- |
| insignificant - rest |


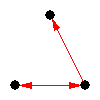
 466


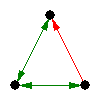
 466


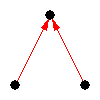
 466


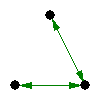
 466


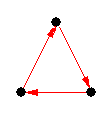


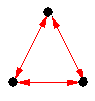


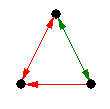


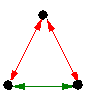


Note:

Red arrows : Transcription Regulation Interaction (TRI)

Green arrows : Protein Protein Interaction (PPI)

*SN : Sub Network

14

| significant - 1 |
| --- |
| insignificant - rest |

174 insignificant

36 insignificant

78 insignificant

Not found

Not found

Not found

Not Found
